# Digital Evolution

**DOI:** 10.1371/journal.pbio.0000018

**Published:** 2003-10-13

**Authors:** Bill O'Neill

## Abstract

In silico experiments reveal how evolution can work--without missing links. What can biologists learn from them?

Rich Lenski decided he was onto a good thing from his very first encounter with digital evolution. It all began when he used the technology in which artificial organisms in the form of computer code evolve independently by self-replicating, mutating, and competing to re-examine an earlier study with bacteria. The original study had contradicted ‘some influential theory’ suggesting that random mutations show a systematic tendency towards synergistic interactions. His digital results, he discovered, matched his organic ones.

‘It's great when these two powerful experimental systems agree, because it suggests some generality about the evolution of genetic architectures', recalls Lenski, professor of microbial ecology at Michigan State University (MSU). ‘But even if the digital and biological realms sometimes come into scientific conflict, it would only lead one to ask why and then probe the relevant factors more deeply’.

## Complex Challenges and the Virtue of Simplicity

**Figure 1:**
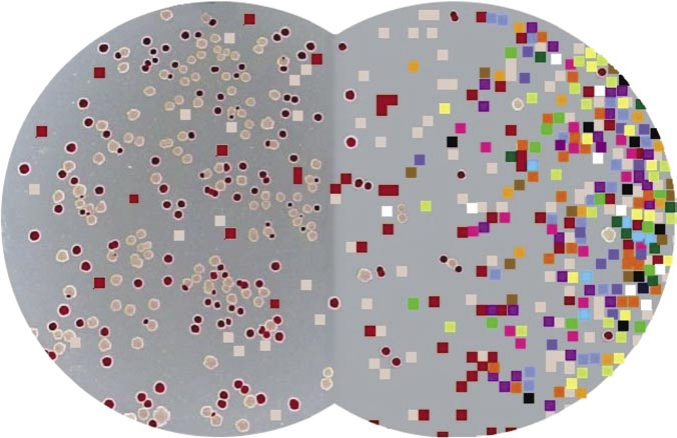
Hybrid Graphic of Petri Dishes with Bacteria Blending into Digital Organisms Lenski spends as much research time with bacteria (left) as he does with digital organisms (right), balancing the strengths and limitations of the two systems in an effort to understand and explain the principles of evolutionary theory. (Hybrid graphic courtesy of Dusan Misevic, Michigan State University.).

He can hardly contain himself. ‘It's a win–win situation, leading towards increased generality, on the one hand, and further experiments to better understand specific outcomes, on the other’. For his part, Lenski has since gone much further with the technology (Box 1; Figure 1) and also soon expects to be announcing results that could broaden digital evolution's appeal even more.

**Box 1. Impossible Evolutionary Experiments**

Richard Lenski is using digital organisms to do ‘impossible’ evolutionary experiments. In one, he says, ‘we test every incipient mutation before it occurs in a population and then allow it or disallow it, depending on its fitness effect, to see how important neutral and deleterious mutations are for long-term adaptation’.

Lenski, professor of microbial ecology at Michigan State University, says his mind boggles at how digital evolution opens up so many avenues for research. ‘I sometimes feel like a kid in a candy store who might starve because he can't make up his mind what he wants’.

These opportunities and, at the other end, the prospect of having too much data to analyse, which Lenski admits is a strange thing for an evolutionary biologist to complain about, enforce a discipline to prioritise and define objectives: ‘What *exactly* is the hypothesis I want to test, and what *exactly* must I measure to test that hypothesis?’

Such enthusiasm for the technology makes it difficult for him to understand why some biologists might dismiss digital evolution as ‘very interesting but with no value’ or turn their backs on it altogether. ‘My own view’, says Lenski, ‘is that something that is very interesting is also worth thinking about and exploring more fully, especially when it offers the opportunity to examine complex problems in greater depth and with more precision than is otherwise possible’.

But he cautions against mistaking his enthusiasm for studying digital organisms as a call to abandon other lines of research. ‘There's obviously much of value for understanding evolution that comes from many different empirical and theoretical perspectives’, he says. ‘That's one reason that evolutionary biology is such a vibrant field right now’.

Lenski still spends as much research time on bacteria as he does on digital organisms. ‘Although it's sometimes frustrating not to be able to devote 100% to each system, each one is so interesting to me that I couldn't bear to drop either of them’. The two systems have different strengths and limitations, which Lenski tries to exploit in his research, he says.

From his laboratory's studies on long-term E. coli populations, he and his colleagues showed earlier this year how they used gene-expression arrays to work backwards to a set of key mutations in a global regulatory gene. More recent work, currently being written up, ‘has led us to some adaptive mutations in several other key loci’, he notes.

As for his digital research using the Avida software system, Lenski acknowledges that speed is an obvious advantage, but not the most significant one. ‘An even more important advantage is the ability to observe the dynamics and dissect the outcomes of evolution with absolute precision. For example, there are no missing links in the digital world’.

Nevertheless, he wryly highlights one shortcoming of Avida: ‘We'll know that we have been successful once the Avidians have evolved the ability to design their own experiments and write the papers without us’.

This is a world where time-scales contract and, above all, where other constraints of ‘wet’ biology have no place.

Earlier this year, he and Chris Adami, who heads the Digital Life Laboratory at the California Institute of Technology (Caltech), published some breathtaking findings from the field. Their collaboration brings together biologists and computer scientists, physicists and philosophers in an artificial world on a quest to understand how evolution works. Though they may still be some way from reaching that objective, their latest advance suggests that they are on the right track.

The research confronts evolutionary theory's long-standing challenge to explain how an organism can develop complex features simply as a result of random mutation and natural selection. The challenge remains a controversial one, too. Supporters of intelligent design, a branch of the creationist movement, promote the notion of ‘irreducible complexity’ as evidence that Darwinian evolution is a flawed theory. The notion purports that a complex feature cannot evolve sequentially from its elements, and must have been designed in one step by some higher intelligence.

Traditional investigations, based on molecular biology and palaeontology, have yielded much evidence about the incremental evolution of the eye or the brain, for instance. But continuing ignorance about many developmental processes and the absence of key fossil records mean that accounts without missing links, to endorse the theory, may never be realised.

Which is what tempted Lenski and Adami to examine the challenge in their virtual world. This is a world where timescales contract and, above all, where other constraints of ‘wet’ biology have no place. ‘It's not just the speed, by any means’, says Lenski. ‘It's also the power to manipulate almost any variable one can imagine, to measure variables with absolute precision, to store information that then allows one to trace back a complex chain of events, and to take evolved organisms and subject them to new sorts of analyses that one might not even have anticipated when first collecting the data’.

It is a place where virtue is made of simplicity. ‘The worlds we're dealing with here are extraordinarily simple compared with the real world’, says Adami. ‘Any of the biochemistry associated with transcription and translation, for example, anything more complex than relatively short viral types of genomes, that's out of our league’, he notes. ‘We can't see transcription and translation because we don't have transcription and translation–-we go right from sequence to function’.

But the principles of evolutionary theory make such restrictions unimportant, he says. ‘Many of the [theory's] predictions don't depend on these little details of molecular biology’, notes Adami. ‘The principles are very, very general, and very simple, and in the end they are mostly responsible for the overall dynamics that you see in these simple systems’. Lenski goes further. These virtual realities, he says, ‘offer us a window into an alternative world, and perhaps even a part of the future of our own, where the fundamental evolutionary mechanisms of mutation and natural selection play out in a novel physical realm’.

Lenski is interested in watching evolution as it happens and has a track record in the study of evolving organic systems, primarily using Escherichia coli. ‘We're making great strides elucidating the precise genetic bases of the adaptation that has occurred during tens of thousands of generations in our long-term E. coli populations’, he reports. ‘Even after more than 30,000 generations in a constant environment, we're still seeing some major phenotypic evolutionary changes’, he adds.

## Evolution in Action

Adami, who also works in theoretical physics at the Jet Propulsion Laboratory at Caltech, has developed a software platform, known as Avida, for research on evolving computer programs, the digital organisms that he terms ‘Avidians’. The second version, Avida 2.0, became available for free public use (http://dllab.caltech.edu/avida/) earlier this year, a decade after work began.

‘I came to Caltech in 1992 on a special fellowship’, he recalls, ‘which basically told me, “You can do whatever you want and we're not going to check on you for three years—just sit there and think of something”’. So he did—and discovered the pioneering work on evolving computer programs by Tom Ray, the computational ecologist who invented the Tierra software system.

‘In a sense, Tom Ray's Tierra was a proof of concept–-he showed that computer programs can evolve, and it was a watershed moment. Without his work, mine wouldn't have existed’, acknowledges Adami. ‘But I wanted this digital life system to be an experimental system just like, let's say, Rich Lenski and E. coli bacteria’.

Adami worked quickly with the help of undergraduates to design and write code and soon had a beta-version ready: ‘Sure, these kids can program’, he laughs. But the programmers were human and errors crept in. The team would run the system overnight and discover ‘weird things’ the next morning: ‘The path of evolution went in a strange way, not because the world dictated it, but because some bug dictated it’, notes Adami. ‘You need to know your system perfectly, at least at the beginning, and that was really the hard part for the next five years’.

On the way, however, the work attracted the attention of Microsoft, the software company, which was eager to know how its designers could evolve computer programs instead of writing them and inevitably introducing bugs, too. Some software already stretches to more than 10 million lines of code, and Microsoft, concerned for its survival as the fittest, foresaw a problem. It predicted programs expanding so much that, sometime between 20 and 50 years into the future, they would reach what Adami calls the ‘complexity wall’, where the number of errors would make them unusable.

The alternative of evolving programs looked like a great idea to Microsoft, especially the way Adami tells it. ‘I know a piece of software that's 3 billion lines of code that controls all our actions’, he says, referring to the human genome. ‘There may be bugs, but they don't lead to a crash. It's very robust programming, with pieces taken from all kinds of different sources, and somehow it works. And the reason why it works is because it was evolved and not written’.

For a year, the Caltech team explored the features of programming languages that make one language more evolvable than another, but moved on when Microsoft's interests switched to more directly applied science and Adami wanted to continue to focus on the fundamental principles underpinning evolution.

Avida was ready to run and beginning to offer a much more versatile platform than Tierra, with advances that have since been honed even further. ‘We can exchange not only the [processor's] instruction set on the fly, we can also change the entire structure of the CPU [central processing unit] on the fly’, says Adami. ‘If you want to test different physics or chemistry, the flexibility of Avida compared with Tierra is like the difference between driving a modern Porsche and a Model-T Ford. They're both cars, but …’

The most important difference, insists Adami, ‘is the possibility of rewards to programs if they accomplish interesting things, in this case computations’. He draws a parallel between the way replicating micro-organisms exploit chemical reactions to yield energy and the way evolving Avidians perform computations to secure extra CPU time. ‘It's a one-to-one analogy’, notes Adami, ‘and the fact that it works so well may tell you something very, very fundamental about the duality between computational chemistries and biochemical chemistries’.

In Adami's collaboration with Lenski to show how complex features can evolve sequentially, the Avidian genome is a circular sequence of instructions in computer code. At the start of its computational existence, an Avidian can only replicate. If it evolves logic functions in the process, however, the system rewards it with energy, in the form of time on the CPU. This reward enables the evolving Avidian to execute instructions that in turn help it to mature to secure more rewards, and so on, to safeguard its future.

The results thrilled the experimenters. Teams at Caltech and MSU were able to trace the genealogy of Avidians, without any missing links, from simple self-replicator through unexpected transitional form to complex performer of many logic functions, with random mutation and natural selection alone responsible for the evolution.

‘Many biologists are delighted to see such a clear demonstration of the evolution from scratch of demonstrably complex features’, says Lenski, ‘and in a way that accords so well with the hypothesis first voiced by Darwin and nowadays supported by a large body of comparative data that complex new features arise by co-opting existing structures that previously served other functions’. He also notes much interest in the way that damaging mutations sometimes proved to be essential stepping stones in the evolution of new functions.

To opponents of evolutionary theory, Lenski is eager to emphasise that the study ‘does not address the origin of life, nor whether the universe itself was designed to allow the evolution of complex organisms. Rather, our study shows that random mutation and natural selection can produce quite complex features, via many pathways, provided that the environment also favours some (but not all) transitional forms, even when the transitional forms are favoured for performing different functions from those that evolve later’.

## The Limits to Truth

For many other biologists, however, digital evolution seems to have very little relevance. One eminent British evolutionary biologist dismissed the research in just eight words, according to the field's godfather, Tom Ray. ‘His comment: “It's just not biology. Period. End of discussion”. That's the whole story right there’, recalls Ray.

Less strident reservations concern the limits on complexity that the virtual world imposes and suspicions about the ability of digital processing to mirror evolutionary principles accurately. For Francisco Ayala, professor of biological sciences at the University of California, Irvine, it appears to be simply a question of trust in the natural world. ‘Computers can give you only what you put in’, he says. ‘With natural models, you're not putting anything in—you're segregating a small region as an aspect of reality’.

There are also more mundane worries over the technical skills needed for the computational operations, a fear acknowledged by Lenski. ‘Computational skills are certainly opening up some exciting new directions [in evolutionary biology]’, he says, ‘but there are of course many other useful skills and fascinating directions’. At Caltech, meanwhile, Adami's team is trying to make Avida easier to use, backed by the National Institutes of Health's first-ever funding for digital-life work.

Misunderstandings about the technology arise over whether the research is an ‘instance’ or a ‘model’ of evolution, suggests Ray, who now divides his time between the Advanced Telecommunications Research Laboratories in Kyoto and the University of Oklahoma, where he holds chairs in zoology and computer science. ‘I never intended [Tierra] as a model, but that's the way a lot of people saw it because they weren't really prepared for this new idea, this different perspective of another instance of life’, he says. ‘They had a more traditional view of what you do with a computer, which is that you send e-mail, you process things, and you make models’.

Levels of veracity determine limits of extrapolation, says Ray. ‘Digital evolution is an abstraction, and it's not going to be able to tell us what humans will evolve into or why dinosaurs went extinct or what will be the next emerging disease…. You need the whole planet to do that kind of modelling’. But once you appreciate the constraints, ‘it's a phenomenally good tool, because it's evolution in a bottle. You can instrument it 100%’, he notes. ‘I think Lenski and Adami have done a very good job of developing it that way’. Ray himself is now more interested in genomics and pharmacology and their application in a biologically inspired engineering project to design software agents, or ‘virtual creatures’, as he terms them.

For Lenski, experiments with Avida provide ‘both an “instance” and a “model” of evolution’. He says that ‘populations of the digital organisms really do evolve and adapt, albeit in an unfamiliar physical realm. At the same time, they provide a sort of experimental model for testing and understanding the general principles of evolution’.

And he agrees with Ray that digital evolution is not intended to explain how we got where we are today, ‘in the sense of unravelling which species are more related to which other species, or what organismal features are adaptive for what purposes, and so forth’. The goal, says Lenski, is to examine evolutionary processes and dynamics in greater depth and detail than are otherwise possible. ‘Watching a process as it occurs and being able to probe genetic details and manipulate environmental variables can provide new insights and evidence that one cannot get by comparative studies that typically require one to infer historical processes from present-day patterns’.

## The First Steps to Freedom

Such developments fascinate and enthral Paul Rainey, an evolutionary ecologist, even though he rarely needs any computing power for his research and recognises that digital evolution still lacks an ecological dimension. Rainey, who earlier this year moved from Oxford to become professor of ecology and evolution at the University of Auckland, uses bacterial populations of Pseudomonas fluorescens, which grow from single genotypes in pristine tubes, to test long-standing hypotheses about the causes of ecological diversification. ‘The bottom line is that we're reducing the complexity we see in the real world to a much more manageable level’, he says. ‘The nice thing about bacterial populations is that ecological and evolutionary timescales coincide, so that you can actually see the ecological context of evolutionary change’.

‘It's a phenomenally good tool, because it's evolution in a bottle.’

Rainey, a friend and colleague of Lenski's, would welcome the chance to take advantage of the speed, robustness, and flexibility of digital evolution to further his research, but doubts whether the technology will ever be able to match the performance of his ‘wet’ laboratory. Though his natural model is simple, it remains far too complex to program, he suspects. ‘We try to understand how selection is working in this very complex ecological context, which includes interactions between genotypes and within genotypes and interactions with an environment that is constantly changing’, he says. ‘This sets the scene for selection, and the selective forces are constantly changing…. None of that complexity is really captured in Avida’.

But Rainey is in for a surprise, according to Adami. ‘The pace of development of Avida has accelerated’, he says. ‘More people are working on it because we have bigger grants. And Charles Ofria [who helped to design the software as a postgraduate at Caltech] is doing much of the development at Michigan State [University, where he is now assistant professor of computer science and engineering] with his students’. The result is that Avidians have made their first steps towards sexual freedom within ecologically diverse environments or, more accurately, code recombinations in a multi-niche virtual world.

For almost a decade, says Adami, Avida has been a single-niche world in which every organism in the population sees exactly the same world and only a single species inhabits that world. But Avida has now been expanded, he continues, ‘in such a manner that populations can see different types of worlds and they can adapt independently to different resources’. A research paper is being finalised on how the software is making its first steps towards incorporating the notion of evolutionary ecology. ‘We show what pressures are necessary to make a population that is homogenous branch out and speciate into a stable system’, notes Adami. ‘Now we want to explore recombination, which we've always shied away from.’ With asexual reproduction virtually understood, the researchers are ready to tackle sexual reproduction in the digital world, says Adami. ‘Some people are furiously working at implementing that.’

‘Our goal is not to mimic natural systems in detail, but rather to expand Avida to give digital organisms access to more of the basic processes of life’, says Lenski. ‘Our goal is not so much to endow the ancestral organisms with additional capabilities, but rather we want to see how digital organisms will evolve if they are placed in an altered world where such things as sex and communication are physically possible. I see many years of interesting research along these lines’.

Reflecting on future applications for the research, Lenski suggests it highlights how the traffic in computational biology is now becoming a significant and little recognised twoway exchange. Computer scientists are not only helping biologists to organise and analyse their vast datasets, says Lenski, but ‘biological principles, from evolution and genetics to neurobiology and ecology, are informing computer scientists and engineers in designing software and hardware … and that holds tremendous promise for the future’.
